# To the Editors of the Medical and Physical Journal

**Published:** 1810-02

**Authors:** M. Ward

**Affiliations:** Manchester


### *To the Editors of the Medical and Physical Journal.*

GENELEMEN,

THE report given of M. Girard's Essay on Tetanus Rabiensis, in page 40 of your last Number, leads me to wish that you would have the goodness to notice, in the way you may think most proper, the view which I took, nearly six years ago, viz. in May, 1804, of the *close analogy between Hydrophobia and Tetanus*; as may be seen by referring to page 94, and 108, 9, of my publication; and to the corresponding places in your Journal, where the two diseases will be found *to be almost identified as one and the same*; the same predisposing and proximate cause being assigned to each, as well as that the proximate effect appeared to me to be the same. This will also be further confirmed by referring to a passage in page 137, 8 of my work, beginning with "The valuable facts," and ending with "e morsu animalis rabidi being added as a definition." *In this paper you will find hydrophobia denominated "Tetanus hydrophobia, or tetanus rabidus,* e morsu animalis rabidi being added as a definition." This, I presume, is a satisfactory and demonstrable claim to an actual *priority of nearly six years* in these doctrines and opinions, over M. Girard; which cannot, I think, considering how extensively your Journal has all along been circulated, not merely upon the continent, but in every quarter of the globe, be resolved into a coincidence of opinion. But this I leave to your better judgment, in the hope that you would be kind enough to notice it in your next, or the succeeding Number. The profession at large should also be informed, and I know of no method more likely to give publicity to the fact, than for it to be announced in your Journal; that in a case of hydrophobia, which occurred at the Manchester Infirmary in August last, and is briefly noticed at the bottom of page 181 of my work, *the cold affusion was repeatedly used, not only without any reluctance being manifested by the patient, but with evident relief to some of the most distressing symptoms, particularly the aversion and difficulty attendant upon the swallowing liquids, &c.; so much so as to have induced the patient to have called for it to be reapplied before the appointed time.* But though the symptoms were in some degree mitigated, the disease terminated fatally, having advanced to nearly the last stage at the time of the patient's admission. The reason these circumstances were omitted in my book is, that I imagined the case would have been published by Dr. Bardsley soon after it happened. Understanding, however, that some time may possibly yet elapse before the particulars will be made public, I have thought it proper to communicate this important fact to you, without meaning to enter any farther into the history or treatment of the disease.

Your very laudable and well known zeal in every thing relating to the interests of medicine, convinces me you will excuse this freedom; in which hope I beg to subscribe myself,

Yours, &c.

*Manchester, Jan.* 19, 18'0. M. WARD.

